# Rigid bronchoscopy: a consultant survey

**DOI:** 10.1308/rcsann.2023.0067

**Published:** 2023-10-16

**Authors:** A Mowat, V Balbirsingh, P Sandhar, M Parekh, A Amlani, B Young, N Giblett

**Affiliations:** ^1^ The Royal Wolverhampton NHS Trust, UK; ^2^ Maidstone and Tunbridge Wells NHS Trust, UK

**Keywords:** Bronchoscopy, Survey, Foreign body

## Abstract

**Introduction:**

Inhalation of foreign bodies represents a potentially fatal emergency in both adults and children. Chest x-ray, in isolation, is neither sensitive nor specific. Rigid bronchoscopy represents the gold standard to diagnose and retrieve paediatric foreign bodies. Cases are encountered infrequently, creating anxieties about their management. Little is known about the confidence in, and maintenance of, rigid bronchoscopy skills by ear, nose and throat teams.

**Methods:**

A 15-question survey was completed by 50 practising otolaryngology consultants in England.

**Results:**

Results show that almost 40% of otolaryngology consultants covering rigid bronchoscopy have not performed bronchoscopy in more than 5 years. Consultants raised concerns about the anaesthetic support and the speed of equipment assembly. Questions on clinical practice showed disparities in practice in the same scenario.

**Conclusions:**

The authors advocate addressing many of the issues raised by the study with a greater availability of simulation courses and regular scheduled intradepartmental teaching days for all professionals involved. National guidelines on criteria for transfer to tertiary centres would improve the consistency of practice.

## Introduction

Inhalation of foreign bodies is a leading cause of death in children aged 1 to 3 years.^1^ The inhaled objects vary between age groups and cultures.^2^ Organic material is more commonly aspirated than non-organic objects,^2^ with nuts being the most frequently encountered bronchial foreign body.^3^ A male preponderance is reported in paediatric cases.^4^ The origin of bronchial foreign bodies is almost exclusively by oral ingestion. Although nasal foreign bodies are managed as an aspiration risk, the chance of a rhinolith entering the bronchial tree is negligible.^5^ Early diagnosis and retrieval is associated with lower morbidity.^6,7^ Positively, the complication rate is falling with time.^8^

Such cases can represent diagnostic dilemmas. Chest x-ray in isolation is imperfect. Typical radiological signs of foreign body aspiration are air trapping, atelectasis and distal infection. In an analysis of 83 consecutive patients, the diagnostic accuracy was 67%.^9^ Performing screening radiographs in the decubitus position does not improve sensitivity.^10^ Hence, clinical management is frequently based on subtleties in the history. This is problematic because the majority of patients are below the age of four.^11^ One retrospective analysis found that only 70% of patients with a confirmed foreign body reported a definite history. Cough and breathlessness were the most common symptoms.^12^ Asymptomatic presentation has been described.^13^

Computed tomography (CT) scanning has progressed to become a viable diagnostic adjunct. Images can be reconstructed to produce an internal view of the trachea and the major bronchi in three dimensions (3D).^14^ Studies support the utilisation of the virtual bronchoscopy in select cases.^15^ The modality can be used to avoid negative rigid bronchoscopy, and provide a road map for surgery. CT is not a perfectly sensitive or specific modality. Mucus plugs can produce false-positive results and minute foreign bodies can be missed.^16^ A comparison study has shown that whereas 100% of patients with bronchial foreign bodies had an abnormal CT finding, 10% of patients with tracheal foreign bodies had normal CT results.^17^ There is no consensus as to whether diagnostic bronchoscopy or CT bronchogram is the best next step in cases of suspected inhaled foreign body with a normal chest x-ray.

Even with improvements in clinical imaging, negative bronchoscopies remain an accepted part of clinical practice. In one retrospective analysis of 1,887 paediatric bronchoscopies, 20.9% failed to identify a foreign body.^18^ The complication rate is not negligible. A retrospective review of 82 cases reported a complication rate of 14.6% in both positive and negative bronchoscopies. The most common were bronchospasm and respiratory distress.^19^

The challenge of early diagnosis is shown by case reports of chronic foreign bodies mimicking lung carcinomas,^20^ asthma, tuberculosis or unresolved pneumonia.^21^ If missed, bronchial foreign bodies can re-present with suppurative lung disease.^22^ In recalcitrant cases of bronchial foreign bodies or delayed presentations, pulmonary resection can be necessary.^23,24^

Cases require clear communication between surgeon and anaesthetist. Once the decision to go to theatre is made, a detailed anaesthetic plan should be discussed.^25^ The choice of induction is influenced by the location of the foreign body. There is a theoretical concern of dislodging an unstable partial proximal obstruction causing complete occlusion on initiation of positive pressure ventilation.^26^ Hence, inhalational induction via a facemask or a cautious intravenous induction that maintains spontaneous ventilation is preferred.^27^ In cases that are expected to require deeper insertion of the bronchoscope, patient mobility and airway reflexes must be suppressed. This is achieved by deeper planes of anaesthesia or neuromuscular blocking drugs. This prevents accidental airway trauma secondary to coughing and bucking, ensuring optimal operating conditions.^25^

Anaesthesia can be maintained by inhalational agents delivered via the anaesthetic circuit attached to the bronchoscope side port. Sevoflurane is the preferred anaesthetic agent owing to its pharmacokinetic properties of rapid onset and offset, and its pharmacodynamic effects of bronchodilation and lack of airway irritability.^28^ Gas escape around the bronchoscope, particularly when working in the larger proximal airways, and hypoventilation can lead to inadequate depths of anaesthesia.^25^ High gas flows are used to combat gas escape, leading to the pollution of the operating theatre.^27^

Recently flexible bronchoscopic methods of retrieval have been described. This can be delivered under sedation utilising a laryngeal mask airway. A retrospective analysis in a tertiary referral centre reported 62 cases managed with flexible bronchoscopic techniques. Of the 28 children in whom an airway foreign body was identified, all the foreign bodies were removed. A further 19 children had no foreign body, but did have macroscopic evidence of previous aspiration. There has been reported success from combining both rigid and flexible bronchoscopy techniques.^29^ Flexible scopes offer the advantage of being able to assess more distal airways and help to reduce the rate of negative findings under rigid bronchoscopies.^30^ Diagnostic procedures can be performed under (deep) angio-sedation.^31^

Reported success rates for foreign body removal via flexible bronchoscopy in children are variable. A small single-centre retrospective analysis showed that conversion to rigid bronchoscopy was required in 75% of cases.^32^ This contrasts with a recent, large single-centre retrospective analysis which reported a success rate using flexible bronchoscopy alone of 99.2%.^33^ The remaining 0.8% of cases were removed with the assistance of a rigid bronchoscope. The varying success rate will be influenced by case volume, which impacts operator experience and skill. The possibility of treatment failure or sudden complication during flexible bronchoscopy means there should always be capacity to perform rigid bronchoscopy as a rescue technique.^30,34^

There is no gold standard technique for airway foreign body removal in adults.^35^ Flexible bronchoscopy is increasingly being used. Scopes can be inserted via an endotracheal tube, laryngeal mask or nasally without an airway device in a sedated patient. The latter avoids some of the detrimental effects of general anaesthesia and preserves the cough reflex. Use of conscious sedation is of particular benefit in adults presenting with airway foreign bodies because of an increased prevalence of neuromuscular or neurodegenerative comorbidities that carry risk of perioperative complication.^35^ However, the technique is not advisable in patients presenting with respiratory distress due to the risk of worsening during the procedure.

At the time of writing, even with advances in flexible bronchoscopy, rigid bronchoscopy remains the gold standard for diagnosis and removal of foreign bodies from the airway of children.^30,32,34,36^

The aim of this study is to provide an insight into consultant rigid bronchoscopy practice in England.

## Methods

A 15-question survey was distributed via www.surveymonkey.com. Fifty responses were received between 1 January and 1 March 2022. The average time taken to complete the survey was 3min. Secretaries in trusts across England were contacted by phone inviting them to distribute the survey via email. All respondents were otolaryngology consultants currently practising in England.

## Results

**Figure 1:**
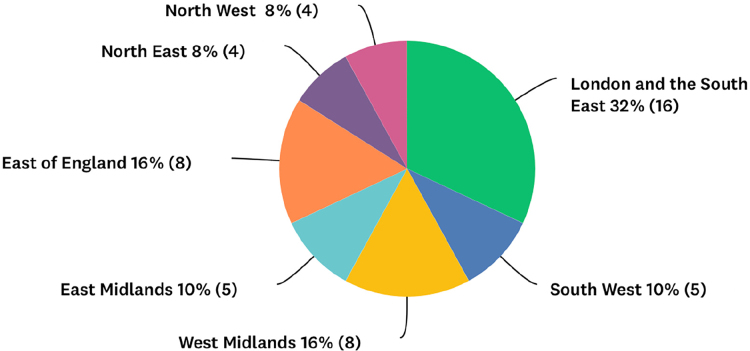
Region of practice of responding consultants

Question 1 asked in which English region the consultant was practising (Figure 1).

Question 2 asked consultants whether they were practising in a teaching or district general hospital (DGH). The majority of respondents (62%) were from DGHs, with 38% from teaching hospitals.

Question 3 asked consultants whether their hospital had a paediatric intensive care unit. Some 30% of respondents had access to level three paediatric care.

**Figure 2:**
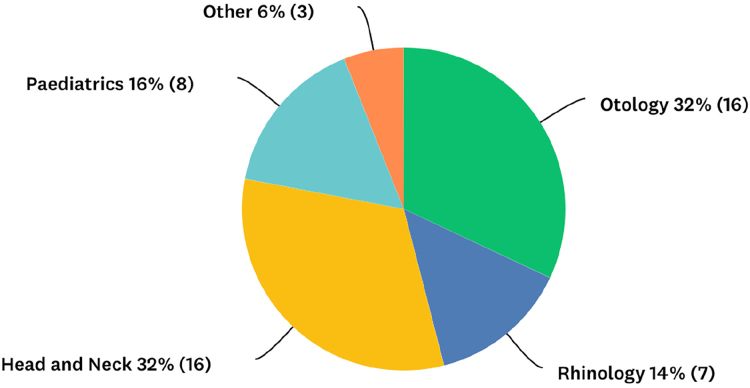
Subspecialty of the consultant responders

Question 4 asked consultants their primary subspecialty (Figure 2).

Question 5 asked consultants the last time had performed paediatric bronchoscopy for the removal of a foreign body in a child. Cumulatively, 20% of consultants had performed paediatric rigid bronchoscopy in the past 6 months and 40% in the past 12 months. However, 38% of respondents had not performed bronchoscopy in the past 5 years.

Question 6 focused on adults, and the responses show even less exposure. Only one respondent had performed a bronchoscopy for removal of a foreign body in an adult in the past 3 months, whereas 34% of respondents had performed adult bronchoscopy for foreign body in the past 12 months.

Question 7 asked consultants about their attendance at bronchoscopy simulation courses. The responses showed that this is inconsistent. Only 20% of consultants reported having attended a simulation course in the past year.

**Figure 3:**
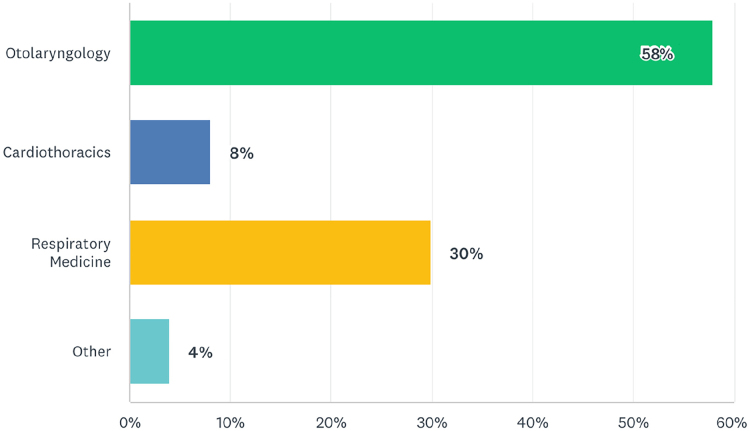
Specialty taking responsibility for adult emergency bronchoscopy

Question 8 asked which specialty took responsibility for emergency adult bronchoscopy in their trust (Figure 3).

Question 9 asked about confidence in equipment assembly. In total, 46% of the consultants reported being extremely or very confident that the equipment would be quickly available; 34% reported being somewhat confident in this regard; and 20% reported being not so confident or not at all confident in the likelihood of all equipment being available to them in a timely fashion.

**Figure 4:**
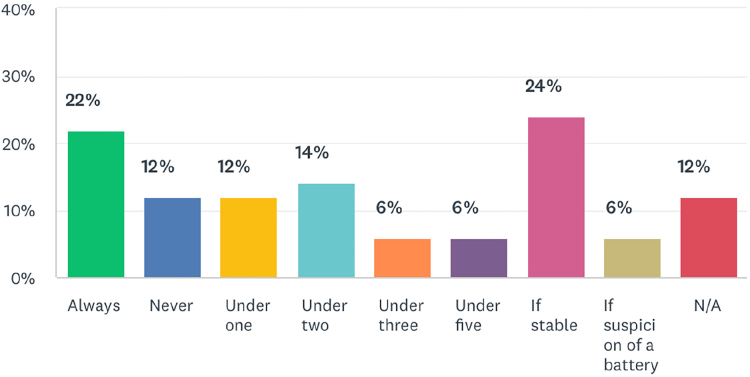
Criteria used by consultants to determine referral to a tertiary centre

Question 10 focused on the criteria for transfer of a case of paediatric bronchial foreign body to a tertiary referral centre. The responses of the 30 DGH consultants are given in Figure 4.

**Figure 5:**
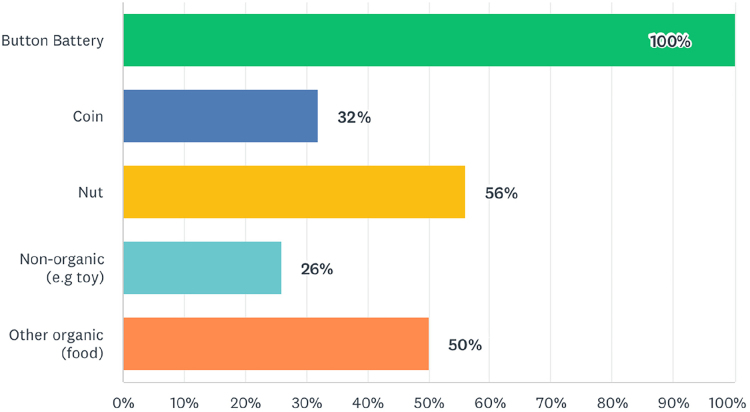
Foreign bodies that consultants would intervene immediately to remove

Question 11 asked consultant responders which foreign bodies would justify immediate intervention clinically when identified, even overnight (Figure 5).

Question 12 assessed a similar clinical scenario in which a stable 6-year-old child is admitted to on-call with a strong history of foreign body inhalation and a normal chest x-ray. Some 50% of respondents suggested that they would proceed to rigid bronchoscopy within 24 hours of admission; 20% said they would obtain a CT thorax before potentially proceeding to surgery. The remaining respondents would monitor the child clinically for 24–48 hours before making a definitive decision. There was consensus that the child should not be discharged.

Question 13 focused on anaesthetic support that would be received during such cases. In total, 24% of otolaryngology consultants expressed great confidence in their anaesthetic colleagues’ competence, and a further 30% of respondents expressed a neutral reply. However, 46%, replied that they had very little confidence in their anaesthetic colleagues.

**Figure 6:**
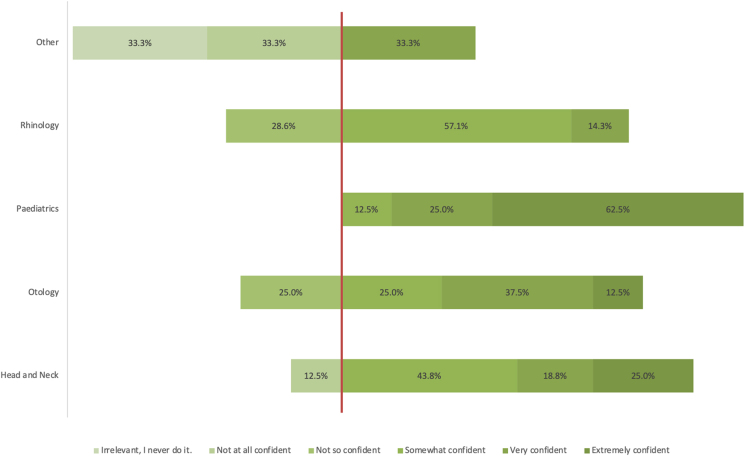
Differing levels of confidence by subspecialty

Question 14 asked consultants to reflect on their own personal confidence levels in performing rigid bronchoscopy. In all, 48% of consultants expressed extremely high or very high confidence levels, and 32% reported being somewhat confident. However, 12% of consultants reported that they were not so confident. A further 6% described themselves as not at all confident in their abilities to perform rigid bronchoscopy (Figure 6).

Question 15 asked about wholistic arrangements for paediatric bronchoscopy in trusts. Only 16% of respondents reported that the current arrangements were excellent; 42% reported that the arrangements were satisfactory; 38% reported that the current arrangements could be improved; and 4% reported that patient safety was threatened.

## Discussion

The first four questions focused on the respondent’s demographics. A range of responses were received with all geographical regions represented. London and the South East had the most respondents, consistent with population breakdown. In England, hospitals are subdivided into peripheral DGHs, which feed into central tertiary referral centres known as teaching hospitals. Paediatric patients are often transferred from DGHs to tertiary referral centres. The division of respondents between DGHs and teaching hospitals (68% vs 32%) is representative of healthcare delivery across England, although exact definitions of what constitutes the hospital subtypes do not exist.^37^ The number of consultants who reported that their hospital had a paediatric intensive care unit was smaller than the number working in a teaching hospital, complicating the distinction further. The responses to question 4 showed a balance of subspecialties, with head and neck being the most represented subspecialty (32%). This fits approximately with the clinical breakdown of consultants by subspecialty in England.^38^ Given the diverse and balanced range of consultants polled, the survey results are probably representative of practice nationally.

The results to questions 5–7 show that consultants are not performing bronchoscopy regularly in adults, children or via simulation. The low rate of adult emergency bronchoscopy displayed in question 6 correlates well with the results of question 8, which asked about the specialty taking responsibility for emergency adult bronchoscopy. Historically, ear, nose and throat (ENT) have taken responsibility for foreign body retrieval. However, there has been a trend towards thoracic surgery or respiratory medicine running the adult on-call service, because bronchoscopy is more commonly encountered in their elective practice. This has further reduced the clinical exposure of the ENT on-call team to foreign body retrieval. Our survey shows 40% of ENT consultants no longer manage emergency adult bronchial foreign bodies. This figure is likely to rise in the upcoming years. The reduction in clinical exposure is not being compensated for by increased attendance at simulation courses. These are now widely offered on the utilisation of bronchoscopy equipment in the emergency setting. Animal models have historically been used to mimic the human thorax. More recently, 3D printing techniques have allowed production of the paediatric airway with greater anatomical accuracy.

The Paediatric ENT Skills Course is available to all ENT consultants in England, and is subsided by ENT UK. Despite its national availability, in our survey only 20% of respondents had attended a simulation course in the past 12 months. Further work is required to assess the ease of course access. This is concerning because it is established that surgical experience correlates positively with good outcomes in foreign body retrieval. A study in China analysed 1,130 children with airway foreign bodies who underwent rigid bronchoscopy; 2.7% had residual foreign bodies confirmed by fibreoptic bronchoscopy. The residual rate of foreign bodies for surgeons with more than 5 years of surgical experience was 1.92% vs 4.25% for those with less.^39^

Because these cases are encountered infrequently, the correct kit can often be difficult to find and assemble in a timely fashion, particularly as the ancillary staff are often minimally exposed to its assembly. The situation is worsened by the significant variation in equipment across units.^40^ Our survey confirms that ENT consultants have ongoing concerns in this regard.

Owing to the anaesthetic complexities of these cases they often represent a source of anxiety for anaesthetists, who also encounter them infrequently. ENT consultants have concerns about the anaesthetic support they would receive in emergency cases of foreign body removal.

The questions on clinical scenarios show inconsistencies in practice. There was consensus that inhalation of button batteries warrants immediate intervention. There is less agreement about other foreign bodies. A majority of consultants would intervene surgically for a nut; 31% would intervene immediately for a coin. The results show that a greater number of consultants would intervene for organic foreign bodies vs non-organic foreign bodies. This reflects the propensity for organic foreign bodies to cause a localised inflammatory response. Referral patterns to tertiary centres were inconsistent; 20% of DGH consultants would always refer paediatric patients to a tertiary centre if there was suspicion of a bronchial foreign body, whereas 12% of DGH consultants would never refer paediatric patients on to a tertiary referral centre. When referral was based on age, there was no consensus on the threshold; the options of 1, 2, 3 and 5 years were all selected by at least one consultant. Thirty-seven per cent of consultants would transfer all stable patients to tertiary referral centres. Such DGH consultants would only take acutely unwell patients to theatre, suggesting a small caseload. Similarly, there was no agreement on whether CT thorax or rigid bronchoscopy represents the next clinical step in a child with a strong history of foreign body inhalation and a normal chest x-ray. The final two questions asked consultants about their own ability, and their trust's delivery of care. Consultants with paediatric subspecialists have the highest confidence levels (Figure 6). Because ENT departments run general on-call services, all subspecialties should be exposed to rigid bronchoscopy with equal frequency. However, it is more common for those with a paediatric bias to encounter rigid bronchoscopy in elective practice. The results of question 15 reflect the concerns raised in earlier parts of the questionnaire. Many consultants reported that their service could be improved or even threatened patient safety.

During ENT training, mastering paediatric airway assessment, particularly addressing paediatric airway obstruction, is a central competence emphasised in the joint committee on surgical training curriculum for attaining certifcate of completion of training recognition.^41^ Displaying adeptness in this fundamental skill mandates completion of ten paediatric airway assessments, along with a formal evaluation to confirm proficiency equivalent to a consultant on their first day of practice. After this phase, surgeons are required to individually maintain their skills in line with the General Medical Council’s Good Medical Practice.^42^ However, when confronting infrequent cases like airway foreign bodies, maintaining consistent surgical exposure becomes challenging. Here, simulation assumes a vital role in ensuring current skills and familiarity with complex techniques and equipment. Implementing interdepartmental simulation sessions becomes crucial, allowing the entire team to collectively refine their abilities and enhance a secure and responsive emergency service.

There is evidence from within a critical care setting that skill decay occurs over time and retraining helps to mitigate this. Regular retraining can help increase ability to adhere to guidelines or perform procedures competently and confidently. One such study analysed paramedics’ ability to place a laryngeal mask airway. Those who had retraining performed significantly better at 6 months. Similar evidence is present with anaesthetists in ‘Can’t intubate, can’t ventilate scenarios’.^43^ Although neither relate to surgical practice directly, they are relevant in practical airway skills; it is therefore likely that skill decay is common in bronchoscopy considering the interval between occurrences. Regular retraining, via simulation courses or virtual reality, will provide mitigation and maintain standards.

## Conclusions

This survey indicates consistent patterns of concern. Bronchoscopy is encountered infrequently by otolaryngologists and engagement with simulation is inconsistent. Disagreement exists in the approach to clinical scenarios. Consultants report little confidence in anaesthetic support or equipment assembly. Increased attendance at interdisciplinary simulation courses or interdepartmental teaching represents a partial solution to these problems. Clearer guidelines on investigation and the transfer of patients would improve consistency of practice.
